# Treatment Strategies for Acute Aortic Dissection With Malperfusion: A Retrospective Study

**DOI:** 10.7759/cureus.65822

**Published:** 2024-07-31

**Authors:** Tomohiro Nakajima, Tsuyoshi Shibata, Kei Mukawa, Shuhei Miura, Ayaka Arihara, Takakimi Mizuno, Keitaro Nakanishi, Yutaka Iba, Nobuyoshi Kawaharada

**Affiliations:** 1 Cardiovascular Surgery, Sapporo Medical University, Sapporo, JPN

**Keywords:** complication, endovascular therapy, operation, malperfusion, aortic dissection

## Abstract

Background: Aortic dissection with malperfusion necessitates emergency surgery and is associated with poor outcomes. Therefore, in this study, we analyzed patients’ treatment courses from the initial management to hospital discharge in cases of acute aortic dissection (AAD) with malperfusion and investigated the risk factors associated with mortality.

Methods: We evaluated cases of AAD with malperfusion treated at our institution over a 16-year period from 2007 to 2022. The primary endpoint was in-hospital mortality. The study's primary outcome measure was mortality during hospitalization. We collected and analyzed data encompassing preoperative patient characteristics, Stanford classification, sites of preoperative malperfusion, surgical techniques employed, and postoperative complications. These variables were examined to identify factors associated with in-hospital mortality.

Results: During the study period, 366 patients were admitted with AAD, 102 of whom had malperfusion. There were 62 men (61%) and 40 women (39%), with a mean age of 64 ± 13 years (range: 28-87 years). According to the Stanford classification, 75 (74%) and 27 (26%) patients had type A and B aortic dissection, respectively, and 29 patients (28%) presented with shock. Preoperative malperfusion sites included the brain, coronary arteries, abdominal viscera, limbs, and spinal cord in 40 (39%), 10 (10%), 34 (33%), 52 (51%), and six (6%) patients, respectively. Eleven (11%) patients required immediate intervention in the emergency department. The treatments administered to the patients were as follows: ascending aortic replacement, 30 (29%) patients; aortic arch replacement, 34 (33%) patients; root replacement, three (3%) patients; thoracic endovascular aortic repair (TEVAR), 12 (12%) patients; non-anatomic bypass, five (5%) patients; and conservative management, five (5%) patients. In-hospital mortality occurred in 23 (23%) patients. Multivariate logistic regression analysis identified preoperative coronary malperfusion as an independent risk factor of mortality.

Conclusion: Preoperative coronary malperfusion is an independent risk factor for in-hospital mortality in patients with AAD presenting with malperfusion.

## Introduction

Aortic dissection is a life-threatening condition, and malperfusion is one of the most serious complications. Malperfusion refers to the impaired blood supply to major organs caused by an abnormal flow distribution between the true and false lumens created by the dissection flap [1]. This can lead to ischemia of the vital organs, such as the brain, coronary arteries, intestines, kidneys, and limbs, potentially resulting in severe clinical symptoms and functional impairments [2-4]. Symptoms vary depending on the affected organ and include stroke or altered consciousness due to cerebral ischemia, abdominal pain, gastrointestinal bleeding from mesenteric ischemia, pain, and motor deficits from limb ischemia [5]. The occurrence of malperfusion depends heavily on the progression of the dissection, hemodynamics between the true and false lumens, and the location of the entry tear. Therefore, prompt and accurate diagnosis and therapeutic intervention are crucial, necessitating a deep understanding of the pathophysiology and clinical manifestations of malperfusion [6]. Hence, this study was aimed at reviewing the current knowledge on the pathogenesis of and diagnostic approach and treatment strategies for malperfusion in aortic dissection and discussing future challenges and perspectives in this area [7].

## Materials and methods

Study population

Patients with acute aortic dissection (AAD) who underwent treatment at Sapporo Medical University Hospital were included. This was a retrospective study.

Analyzed factors and the primary endpoint

The primary endpoint was the in-hospital mortality rate. Data on preoperative patient factors, Stanford classification, preoperative malperfusion site, surgical procedure, and postoperative complications were collected, and factors related to in-hospital mortality were analyzed.

Strategy of AAD

Patients diagnosed with aortic dissection at other facilities are referred to Sapporo Medical University Hospital for surgical management. Upon arrival, contrast-enhanced computed tomography (CECT) was performed to confirm the diagnosis. Emergency surgical repair was performed in the patients with Stanford type A aortic dissection. In cases of Stanford type B dissection, conservative medical management is the standard approach in the absence of malperfusion. However, malperfusion necessitates emergency surgical interventions.

Definitions of malperfusion

Aortic dissection is a life-threatening condition and malperfusion is one of the most serious complications. Malperfusion refers to inadequate or insufficient blood supply to major organs due to an abnormal flow distribution between the true and false lumens formed by aortic dissection. Specifically, it involves reduced or obstructed blood flow to vital organs (such as the brain, coronary arteries, intestines, kidneys, and limbs), leading to organ dysfunction.

Malperfusion was diagnosed by considering the clinical presentation, imaging findings, and laboratory test findings. Symptoms may include neurological deficits, such as weakness or numbness (cerebral malperfusion), chest pain (coronary malperfusion), abdominal pain (mesenteric malperfusion), or lower extremity pain and pallor (limb malperfusion). Imaging modalities, such as CT or magnetic resonance imaging (MRI), are crucial for visualizing disrupted blood flow and the site of compromise within the affected vascular territory. Elevated levels of biomarkers such as troponins or D-dimers may aid in localizing the involved vascular bed.

Statistical methods

Continuous variables are reported as mean ± standard deviation values. Categorical variables are presented as raw numbers (percentages) and were compared using the χ2 and Fisher’s exact tests. All calculations were performed using JMP version 17 (SAS Institute Inc., Cary, NC, USA).

## Results

Patient characteristics

In total, 102 patients were enrolled in this study. Their mean age was 64 ± 13 years, and 62 patients (61%) were male. According to the Stanford classification, there were 75 cases (74%) of type A and 27 cases (26%) of type B aortic dissection. Twenty-nine patients (28%) experienced shock. Preoperative malperfusion sites involved the brain in 40 (39%), coronary arteries in 10 (10%), abdominal viscera in 34 (33%), limbs in 52 (51%), and spinal cord in six (6%) patients. Ten patients (10%) required immediate intervention in the emergency department, six (6%) required endotracheal intubation, two (2%) required pericardial drainage, and two (2%) required extracorporeal membrane oxygenation (ECMO).

Operative characteristics

The surgical procedures performed are summarized in Table 1 and included ascending aortic replacement in 30 cases (29%), aortic arch replacement in 34 (33%), root replacement in three (3%), thoracic endovascular aortic repair (TEVAR) in 12 (12%), non-anatomic bypass in five (5%), and conservative management in five (5%).

**Table 1:** Procedure for aortic dissection Categorical data are presented as numbers (%).

Variables	Overall (N = 102)
Procedure	
Aortic root replacement	3 (3)
Ascending aorta graft replacement	30 (29)
Total arch graft replacement	34 (33)
Thoracic endovascular aortic repair	12 (12)
Non-anatomical bypass	5 (5)
Preserved treatment	5 (5)

Postoperative findings

In-hospital mortality occurred in 23 (23%) patients. Multivariate logistic regression analysis was performed to identify factors associated with mortality. Preoperative cardiac malperfusion was identified as an independent risk factor for mortality (odds ratio 40, p < 0.01). The results are summarized in Table 2.

**Table 2:** Odds ratios related to malperfusion frequency and in-hospital mortality in acute aortic dissection

	Cerebral	Coronary	Colon	Extremities	Spinal
Malperfusion	40 (39%)	10 (10%)	34 (33%)	52 (51%)	6 (6%)
Odds ratio	3.1	40	2.5	1.4	2.2
95% CI	0.77-12.2	5.4-299	0.60-10.0	0.46-4.34	0.98-1.10
p-value	0.10	<0.01	0.21	0.55	0.19

## Discussion

Aortic dissection is a severe condition characterized by a tear in the intima of the aortic wall that allows blood to flow into the media, thereby separating the intimal and medial layers. The primary presenting symptoms are acute chest and back pain, with hypertension and atherosclerosis as the main risk factors [8]. As the dissection progresses, it may lead to life-threatening complications such as organ ischemia or aortic rupture. Diagnosis typically involves CT or echocardiography [9]. Treatment modalities, including medical management or surgical intervention, are selected based on the location of the dissection and complications. Early diagnosis and appropriate therapeutic intervention are crucial for improving the prognosis [10].

Organ ischemia in aortic dissection, once initiated, tends to progressively worsen over time. There are two primary approaches for alleviating organ ischemia: one involves prioritizing the treatment of aortic dissection through central repair and the other focuses on initiating local perfusion first [11,12]. Organs are particularly susceptible to irreversible ischemic damage without immediate restoration of local perfusion including the brain, heart, and superior mesenteric arteries [13]. Currently, treatment strategies vary among institutions [14].

In the treatment of aortic dissection, central repair involves closing the entry site of the dissection under general anesthesia by using artificial graft replacement or stent grafting. Even with the most expeditious treatment, this process typically requires two to three hours from hospital arrival to completion. Recent reports have described the use of endovascular techniques to address coronary perfusion deficits [10]. Additionally, intentional fenestration between the true and false lumens has been reported as a method of alleviating intestinal ischemia [15].

Results of this study and a literature review

This single-center study examined patients with AAD complicated by malperfusion deficits. Of the 366 patients with aortic dissection admitted during the study period, 102 (27.9%) presented malperfusion deficits. Risk factor analysis of in-hospital mortality revealed that coronary perfusion deficit was a significant contributing factor. Notably, no cases in this series required the management of malperfusion deficits prior to aortic repair [11]. This can be primarily attributed to the institution's policy of transferring patients requiring emergency surgery to nearby facilities when they cannot be accommodated internally. Additionally, the lack of experience in managing malperfusion deficits before aortic repair has led to a preference for prioritizing vascular interventions (Table 3) [16].

**Table 3:** List of the literature on aortic dissection and malperfusion

No.	Author’s Name	Title of the Paper	Target of Organs With Malperfusion
1	Yang et al., 2019 [1]	Managing patients with acute type A aortic dissection and mesenteric malperfusion syndrome: a 20-year experience	Cerebral
2	Tong et al., 2022 [11]	Coronary malperfusion secondary to acute type A aortic dissection: surgical management based on a modified Neri classification	Coronary
3	Morisaki et al., 2015 [7]	Delayed intestinal ischemia after surgery for type A acute aortic dissection	Colon
4	Norton et al., 2022 [5]	Treating lower extremity malperfusion syndrome in acute type A aortic dissection with endovascular revascularization followed by delayed aortic repair	Extremities
5	Elshony et al., 2021 [4]	Spinal cord ischemia secondary to aortic dissection: case report with literature review for different clinical presentations, risk factors, radiological findings, therapeutic modalities, and outcome	Spinal cord

Limitations

This was a single-center, retrospective analysis, so there may be some bias in the number of patients.

## Conclusions

We analyzed the factors associated with mortality among patients who developed malperfusion deficits prior to surgery for aortic dissection. We found that coronary perfusion deficit was a significant factor contributing to mortality. Notably, at our institution, there were no cases in which reperfusion was initiated for organs with perfusion deficits; instead, the aortic repair was prioritized in all cases.
